# Myogenic Disease and Metabolic Acidosis: Consider Multiple Acyl-Coenzyme A Dehydrogenase Deficiency

**DOI:** 10.1155/2019/1598213

**Published:** 2019-12-21

**Authors:** A. Dernoncourt, J. Bouchereau, C. Acquaviva-Bourdain, C. Wicker, P. De Lonlay, C. Gourguechon, H. Sevestre, P.-E. Merle, J. Maizel, C. Brault

**Affiliations:** ^1^Intensive Care Unit, Amiens University Medical Center, F-80000 Amiens, France; ^2^Reference Center for Hereditary Metabolic Diseases, Necker Hospital, Public Hospitals of Paris, F-75019 Paris, France; ^3^Reference Center for Hereditary Metabolic Diseases (Eastern France), Department of Pathology and Biology, Hospices Civil de Lyon, F-69500 Bron, France; ^4^Department of Internal Medicine and Endocrinology, Abbeville Hospital, F-80100 Abbeville, France; ^5^Department of Pathological Anatomy and Cytology, Amiens University Medical Center, F-80000 Amiens, France; ^6^Functional Investigation of the Nervous System Unit, Amiens University Medical Center, F-80000 Amiens, France

## Abstract

**Background:**

Multiple acyl-coA dehydrogenase deficiency (MADD) is a rare, inherited, autosomal-recessive disorder leading to the accumulation of acylcarnitine of all chain lengths. Acute decompensation with cardiac, respiratory or hepatic failure and metabolic abnormalities may be life-threatening.

**Case Presentation:**

A 29-year-old woman presented with severe lactic acidosis associated with intense myalgia and muscle weakness. The clinical examination revealed symmetric upper and lower limb motor impairment (rated at 2 or 3 out of 5 on the Medical Research Council scale) and clear amyotrophy. Laboratory tests had revealed severe rhabdomyolysis, with a serum creatine phosphokinase level of 8,700 IU/L and asymptomatic hypoglycemia in the absence of ketosis. Electromyography revealed myotonic bursts in all four limbs. The absence of myositis-specific autoantibodies ruled out a diagnosis of autoimmune myositis. Finally, Acylcarnitine profile and gas chromatography–mass spectrometry analysis of organic acids led to the diagnosis of MADD. A treatment based on the intravenous infusion of glucose solutes, administration of riboflavin, and supplementation with coenzyme Q10 and carnitine was effective. Lipid consumption was strictly prohibited in the early stages of treatment. The clinical and biochemical parameters rapidly improved and we noticed a complete disappearance of the motor deficit, without sequelae.

**Conclusion:**

A diagnosis of MADD must be considered whenever acute or chronic muscle involvement is associated with metabolic disorders. Acute heart, respiratory or hepatic failure and metabolic abnormalities caused by MADD may be life-threatening, and will require intensive care.

## 1. Background

Multiple acyl-coenzyme A dehydrogenase deficiency (MADD), also known as glutaric aciduria type II, is a disorder of fatty acid oxidation. Although MADD is usually diagnosed during the neonatal period, late-onset forms may sometimes only be revealed during adulthood. Here, we report on the case of a 29-year-old woman hospitalized in the intensive care unit for motor impairment of the four limbs and presenting with rhabdomyolysis, severe lactic acidosis, and hypoketotic hypoglycemia. The case illustrates the diagnostic and therapeutic strategies for managing an acute decompensation of MADD.

## 2. Case Presentation

A 29-year-old woman with an unremarkable personal medical history (including two complication-free pregnancies) was admitted to our intensive care department for the treatment of severe lactic acidosis related to myopathy. For the previous 9 months, the woman had experienced intense myalgia and muscle weakness. These signs and symptoms were initially limited to the quadriceps (she was unable to squat or climb stairs) but extended progressively to all four limbs—leading to major functional disability. Out-of-hospital laboratory tests had revealed moderate rhabdomyolysis, with a serum creatine phosphokinase level of 500 IU/L. The initial assessment was negative: there was no evidence of a drug-related etiology, thyroid disorder, viral infection (with negative serological tests for HIV, HBV, and HCV) or bacterial infection (with a negative serological test for Lyme disease).

Due to a major deterioration in the woman's general health status (with an estimated weight loss since symptom onset of 20 kg), aggravation of the motor impairment (preventing her from standing and confining her to a wheelchair), and the recent onset of a swallowing disorder, the patient was referred to our medical center's emergency department by her family physician. The clinical examination revealed symmetric upper and lower limb motor impairment (rated at 2 or 3 out of 5 on the Medical Research Council scale) and clear amyotrophy. Although neither sensory impairment nor involvement of the cranial pairs was noted, some deep tendon reflexes were absent. The laboratory test results highlighted uncompensated metabolic acidosis (pH: 7.28, pCO_2_: 28 mmHg, HCO3^−^: 14 mmol/L) due to hyperlactatemia (11 mmol/L). The rhabdomyolysis had worsened, with a serum creatine phosphokinase level of 8,700 IU/L and asymptomatic hypoglycemia (1.1 mmol/L) in the absence of ketosis. No inflammatory syndromes, renal impairments, and or electrolyte balance disorders were apparent. A blood test for elevated beta-hCG was negative. There were no cardiac impairments: the transthoracic ultrasound, electrocardiogram and troponin assay results were unremarkable, as was a whole-body computed tomography scan.

**Figure 1:**
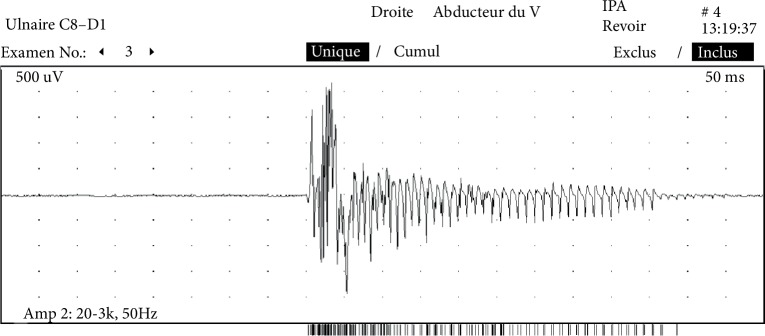
Electromyogram of the right 5th finger. A surface electromyogram, showing myotonic bursts in the abductor of the right 5th finger.

**Figure 2:**
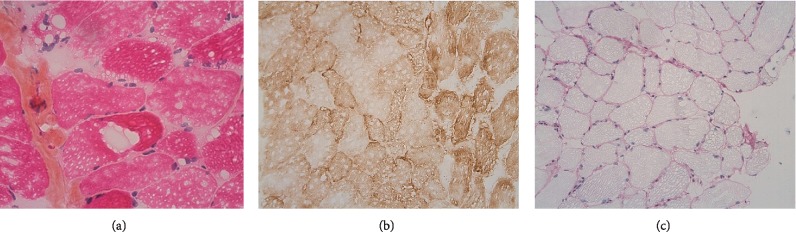
Pathology assessment of a muscle biopsy. (a) Stain:hematein eosin saffron; magnification: ×40. (b) Histochemical analysis of cytochrome oxidase; magnification: ×20. (c) Stain: periodic acid Schiff; magnification: ×20.

Given this context, the patient was transferred to our intensive care unit. Electromyography revealed myotonic bursts in all four limbs (Figure 1). The absence of myositis-specific autoantibodies ruled out a diagnosis of autoimmune myositis. The patient's parents told us that their younger daughter had died suddenly in the postpartum period at the age of 22, and that she was being monitored for a possible beta oxidation disorder. A complete metabolic assessment was carried out, for a suspected deficiency in glucose-lipid metabolism decompensated by the prolonged fasting induced by a swallowing disorder. A chromatographic analysis of urine organic acids and plasma acylcarnitine suggested the presence of multiple acyl-coA dehydrogenase deficiency (MADD) (Tables 1(a) and 1(b)). Moreover, the venous blood ammonia level was normal, and a chromatographic analysis of amino acid profile was normal. A histologic assessment of a muscle biopsy showed clusters of sometimes confluent vacuoles within the muscle fibers; this observation was consistent with lipid overload in striated muscle (Figure 2).

**Figure 3:**
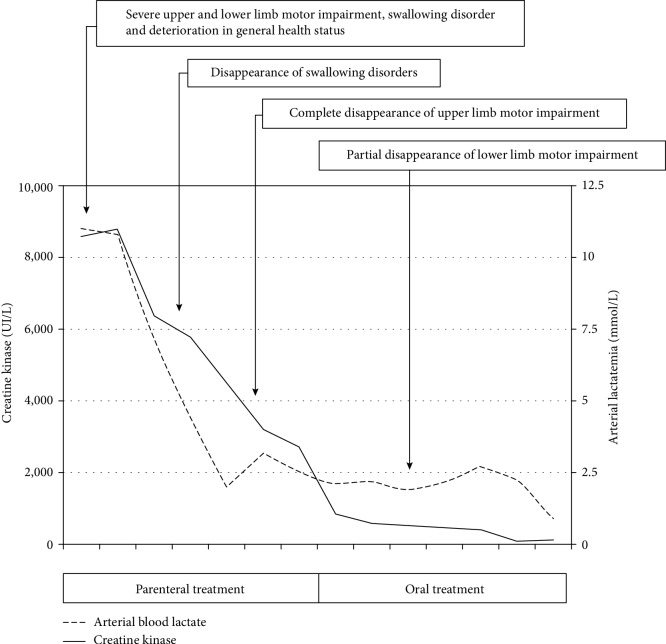
Changes in clinical and laboratory parameters during treatment.

Following a discussion with the medical team at the reference center for hereditary metabolic diseases (Necker Hospital, Paris, France), an appropriate treatment was initiated: the intravenous infusion of 10% glucose solution (3 liters over 24 hours) and supplementation with L-carnitine (6 g per 24 h), L-glycine (2 g every 6 h), and riboflavin (500 mg every 8 h). Initially, lipid consumption was formally contraindicated. The clinical and biochemical parameters improved (Figure 3); the rapid disappearance of the swallowing disorder enabled the resumption of oral feeding (a low fat diet, with lipids accounting for no more than 5–10% of the recommended total energy intake). After normalization of the electrolyte balance and glycemia, oral treatment was initiated using the above-mentioned doses and the addition of coenzyme Q10 (200 mg three times a day). The patient's meals were enriched with omega 3 fatty acids (walnut oil). On day 14 postadmission to the ICU, the patient was transferred to a general medical ward and then to a rehabilitation center. On day 40, the patient returned home. There were no sequelae. The diagnosis of MADD was later confirmed by the identification of heterozygosity for the two inherited mutations (c.1448C>T (exon 11) and c.1601C>T (exon 12)) in the *ETFDH* gene coding for electron transfer flavoprotein dehydrogenase (also known as electron transfer flavoprotein-ubiquinone oxidoreductase).

## 3. Discussion

**Figure 4:**
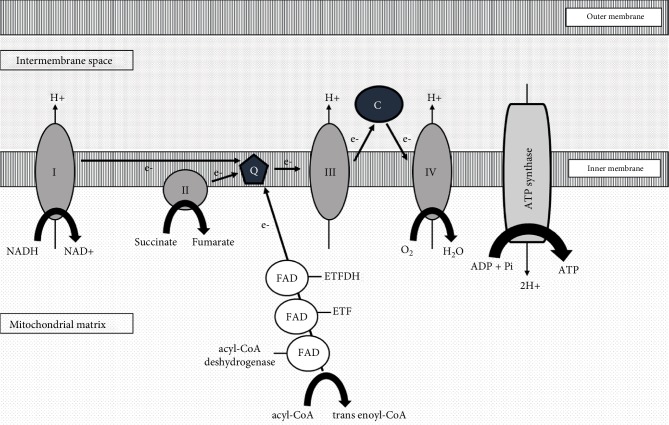
Simplified illustration of the mitochondrial respiratory chain. The respiratory chain is an electron transport chain that oxidizes reduced coenzymes (such as NADH) resulting from the degradation of organic compounds. It is composed of four protein complexes (I–IV) located in the inner mitochondrial membrane. The complexes are associated with two cofactors, coenzyme Q10 (Q) and cytochrome (C). This oxidation provides the energy required for proton transfer from the matrix to the mitochondrial intermembrane space. The resulting electrochemical gradient provides the energy needed for ATP synthase to phosphorylate ADP to ATP. The enzymes' electron transfer flavoprotein (ETF) and ETF dehydrogenase (ETFDH) catalyze the transfer of electrons produced by fatty acid oxidation from acyl-coenzyme A dehydrogenase to coenzyme Q10, using flavin adenine dinucleotide (FAD) as a cofactor.

Multiple acyl-coA dehydrogenase deficiency is a rare, inherited, autosomal-recessive disorder caused by the lack of ETFDH in the mitochondrial flavoprotein chain [1–4]. The ETF/ETFDH serves as an electron transfer pathway to conduct electrons, produced by fatty acid and amino acid oxidation, from the different mitochondrial flavoprotein dehydrogenases to the terminal respiratory chain (Figure 4) [5–7]. A mitochondrial flavoprotein deficiency impairs ATP biosynthesis and gluconeogenesis, and leads to the accumulation of acylcarnitine of all chain lengths. A histologic assessment highlighted an excessive accumulation of lipid in muscle tissue, the liver and the myocardium [3, 8].

There are three MADD phenotypes, which differed in severity and age at symptom onset (Table 2) [8–10]. Late-onset MADD is characterized by broad clinical and genetic heterogeneity - even within the same family [3]. The main symptom is chronic myalgia, along with exercise intolerance or muscle weakness in some cases. Cardiomyopathy, liver deficiency, and respiratory failure due to a restrictive lung disease may also appear [8, 11]. In addition to these chronic manifestations, 30% of patients with MADD are affected by acute decompensation (also referred to as a “metabolic crisis”) [2]. These crises are often triggered by catabolic stress, e.g. in a context of prolonged fasting, sepsis, or pregnancy [2]. The main signs are the exacerbation of muscle symptoms and rhabdomyolysis [10, 11]. Muscle impairment may progress rapidly and can mimic Guillain Barré syndrome, myositis, or myasthenia gravis [12, 13]. Acute respiratory failure may be caused by involvement of the diaphragm or the accessory respiratory muscles [10, 11]. Cases of cardiomyopathy and sometimes fatal cardiac arrhythmia have been reported, especially in children [14, 15]. Lastly, consciousness disorders related to hyperammonemic encephalopathy may appear, and are sometimes linked to acute hepatic insufficiency [9]. With regard to biochemical parameters, nonketonic hypoglycemia and severe lactic acidosis are frequently observed [1, 8]. These serious and sometimes inaugural complications of MADD can be life-threatening in the absence of appropriate treatment; in most cases, they require intensive care [2, 16].

A diagnosis of MADD should be considered when a chromatographic urine organic acid analysis shows elevated levels of dicarboxylic, glutaric, ethylmalonic, and lactic acids. The plasma acylcarnitine profile shows the accumulation of short-, medium- and long-chain acylcarnitines (C4–C18). However, normal results in these analyses do not preclude a diagnosis of MADD, especially if the analyses are performed outside the acute decompensation period [8–11]. Electromyography typically reveals a myogenic pattern, whereas a muscle biopsy usually shows lipidosis within type I muscle fibers [10–12]. In more than 90% of cases, the patient carries one or more homozygous or heterozygous mutations within the 13 exons of the *ETFDH* gene, whereas the genes encoding the *α* and *β* subunits of the electron transfer flavoprotein are mutated in, respectively, 5% and 2% of cases only [10, 17, 18]. Flavoprotein mutations can affect protein folding and assembly or can affecting enzymatic activity leading to compromised fatty acid oxidation. A family history of sudden death, especially in the neonatal period, or cardiomyopathy can suggest the can guide the MADD diagnosis.

In cases of MADD with severe metabolic abnormalities, the treatment is based on the intravenous infusion of glucose solutes to promote anabolism and prevent lipolysis [19]. Intravenous or oral administration of riboflavin (vitamin B2, a water-soluble vitamin required for the synthesis of flavin adenine dinucleotide) will be effective in some patients [9, 19]. Supplementation with coenzyme Q10 and carnitine is also necessary because secondary deficiencies are common [20]. Lipid intake is prohibited in the early stages of treatment [19]. The clinical outcome is often positive, although muscle-related or hepatic sequelae may persist—especially when severe metabolic crises have been treated late [2, 12]. Patients should continue long-term oral supplementation and follow a low-fat diet (with fat accounting for 10–15% of the daily calorie intake) that is rich in proteins and complex carbohydrates. Protein intake should be limited to the theoretical value of 1 g/kg/d, given the risk of hyperammonemia related to ETFDH's role in the branched-chain amino acid metabolism. Patients should especially avoid prolonged fasting because catabolism is a major risk factor for decompensation [19]. Patient education, greater awareness among physicians, and family screening are also essential for the optimal management of MADD. Patients should be cared for by a metabolic disease specialist.

## 4. Conclusion

A diagnosis of MADD must be considered whenever acute or chronic muscle involvement is associated with metabolic disorders. Acute heart, respiratory, or hepatic failure and metabolic abnormalities caused by MADD may be life-threatening, and will require intensive care.

## Figures and Tables

**Table:** (a) Gas chromatography–mass spectrometry analysis of organic acids

Organic acid	Qualitative or quantitative assay result (mmol/mmol creatinine)
Lactic acid	**Elevated (124.1)**
Glycolic acid	10.8
3-Hydroxypropionic acid	Present
3-Hydroxybutyric acid	19.9
3-Hydroxyisovaleric acid	Present
Methylmalonic acid	Present
Ethylmalonic acid	**Elevated (26.0)**
Succinic acid	Present
Methylsuccinic acid	**Elevated **(value not reported)
Fumaric acid	**Elevated (17.1)**
Glutaric acid	**Elevated (164.6)**
Adipic acid	**Elevated (707.3)**
Suberic acid	**Elevated (147.5)**
Sebacic acid	Missing
Cis-4-octene dioic acid	Present
Cis-4-decene dioic acid	Present
5-Hydroxyl caproic acid	**Elevated **(value not reported)
3-Hydroxydecane dioic acid	Present
3-Hydroxydecene dioic acid	Present
3-Hydroxydodecane dioic acid	Present
3-Hydroxydodecene dioic acid	Present
Isobutyryl glycine	Present
Butyryl glycine	Present
2-Methyl butyryl glycine	Present
Isovaleryl glycine	**Elevated (178.2)**
Hexanoyl glycine	**Elevated (28.2)**
Suberyl glycine	Present
4-Hydroxyphenyllactic acid	**Elevated (212.8)**
2-Hydroxyglutaric acid	**Elevated (120.0)**

**Table:** (b) Acylcarnitine profile

Acylcarnitine	Assay result (*μ*mol/L)
C0: Free L-carnitine	**↓ 3.7**
Total C0: Total L-carnitine	**↓ 18.7**
Free carnitine/total carnitine	**↓ 0.20**
C2: Acetyl carnitine	3.34
C3: Propionyl carnitine	0.07
C4: Butyryl/isobutyryl carnitine	**↑ 0.71**
C5: Isovaleryl/2-methylbutyryl carnitine	**↑ 0.55**
C6: Hexanoyl carnitine	0.09
C8: Octanoyl carnitine	**↑ 0.24**
C10: Decanoyl carnitine	**↑ 0.47**
C12: Dodecenoyl carnitine	**↑ 0.71**
C14: Tetradecanoyl carnitine	**↑ 1.05**
C16: Palmitoyl carnitine	**↑ 5.09**
C18: Stearyl carnitine	**↑ 2.65**

**Table 2:** Description of the different phenotypes of DMAD.

Phenotype	Age of onset	Main clinical manifestations	Laboratory abnormalities
I	Neonatal	Congenital malformations such as facial dysmorphism, brain malformation, renal dysplasia, cardiomyopathy, arrhythmia, and hepatomegaly	Non-ketotic hypoglycemia
		Hypotonia	
		Fatal outcome in a few days	
			Severe metabolic acidosis

II	Neonatal	No congenital malformations	Hypoketotic hypoglycemia
		Cardiomyopathy and arrhythmia	Moderate metabolic acidosis
		Hypotonia	
		Fatal outcome in a few weeks without treatment	

III	Childhood or adulthood	Myalgia, muscle weakness, fatigability	
		Hepatomegaly	
		Cardiomyopathy	
		Chronic respiratory failure due to restrictive lung disease	
		Peripheral neuropathy	

Acute decompensation (metabolic crisis)		Worsening of chronic muscle symptoms	Metabolic acidosis
		Vomiting	Hypoketotic hypoglycemia
		Acute respiratory failure	Rhabdomyolysis
		Acute liver failure	Hepatic cytolysis
		Encephalopathy	Hyperammonemia
		Can occur regardless of the phenotype and age of the patient	
		Life-threatening prognosis in the absence of appropriate treatment	

## Data Availability

The datasets used and/or analyzed during the current study are available from the corresponding author on reasonable request.
